# The D113N mutation in the RING E3 ubiquitin protein ligase gene is not associated with ex vivo susceptibility to common anti-malarial drugs in African *Plasmodium falciparum* isolates

**DOI:** 10.1186/s12936-018-2252-2

**Published:** 2018-03-12

**Authors:** Mathieu Gendrot, Francis Tsombeng Foguim, Marie Gladys Robert, Rémy Amalvict, Joel Mosnier, Nicolas Benoit, Marylin Madamet, Bruno Pradines, the French National Reference Centre for Imported Malaria Study Group, V. Augis, D. Basset, P. Bastien, F. Benoit-Vical, A. Berry, P. Brouqui, M. Cividin, P. Delaunay, L. Delhaes, M. Drancourt, T. Gaillard, A. Genin, E. Garnotel, E. Javelle, C. L’Ollivier, M. Leveque, D. Malvy, P. Marty, M. Mechain, G. Ménard, P. Millet, P. Minodier, A. Mottard, P. Parola, R. Piarroux, C. Pomares-Estran, M. -C. Receveur, A. Robin, E. Sappa, H. Savini, F. Simon, Y. Sterkers, C. Surcouf, E. Varlet, A. Wolff

**Affiliations:** 1 Unité Parasitologie et entomologie, Département des maladies infectieuses, Institut de recherche biomédicale des armées, Institut hospitalo-universitaire (IHU) Méditerranée Infection, 19-21 Boulevard Jean Moulin, 13005 Marseille, France; 2 Aix Marseille Univ, IRD, AP-HM, SSA, VITROME, IHU-Méditerranée Infection, Marseille, France; 3 Centre national de référence du Paludisme, Institut hospitalo-universitaire (IHU) Méditerranée Infection, Marseille, France

**Keywords:** Malaria, *Plasmodium falciparum*, Anti-malarial drug, In vitro, Resistance, Molecular marker, RING E3 ubiquitin-protein ligase gene

## Abstract

**Background:**

*Plasmodium falciparum* resistance to artemisinin-based combination therapy has emerged and spread in Southeast Asia. In areas where artemisinin resistance is emerging, the efficacy of combination is now based on partner drugs. In this context, the identification of novel markers of resistance is essential to monitor the emergence and spread of resistance to these partner drugs. The ubiquitylation pathway could be a possible target for anti-malarial compounds and might be involved in resistance. Polymorphisms in the E3 ubiquitin-protein ligase (PF3D7_0627300) gene could be associated with decreased in vitro susceptibility to anti-malarial drugs.

**Methods:**

*Plasmodium falciparum* isolates were collected from patients hospitalized in France with imported malaria from a malaria-endemic country from January 2015 to December 2016 and, more particularly, from African French-speaking countries. In total, 215 isolates were successfully sequenced for the E3 ubiquitin-protein ligase gene and assessed for ex vivo susceptibility to anti-malarial drugs.

**Results:**

The D113N mutation in the RING E3 ubiquitin-protein ligase gene was present in 147 out of the 215 samples (68.4%). The IC_50_ values for the ten anti-malarial drugs were not significantly different between the wild-type and mutant parasites (p values between 0.225 and 0.933). There was no significant difference in terms of the percentage of parasites with decreased susceptibility between the D113 wild-type and the 133N mutated *P. falciparum* strains (p values between 0.541 and 1).

**Conclusion:**

The present data confirmed the absence of the association between polymorphisms in the RING E3 ubiquitin-protein ligase gene and the ex vivo susceptibility to common anti-malarial drugs in African *P. falciparum* isolates.

## Background

*Plasmodium falciparum* resistance to most anti-malarial drugs has emerged in Southeast Asia and spread to Africa [1, 2]. Since 2005, the World Health Organization (WHO) has recommended artemisinin-based combination therapy (ACT) as first-line treatment for uncomplicated malaria and intravenous artesunate for the treatment of severe malaria. However, artemisinin derivative resistant *P. falciparum* strains have emerged in western Cambodia, Myanmar and Thailand and throughout Southeast Asia [3–8]. This resistance has resulted in a delayed parasite clearance in patients treated with artesunate monotherapy or ACT therapy [3, 4]. In areas where artemisinin resistance is emerging, combination efficacy is based now on partner drugs. In this context, the discovery of novel markers of resistance is essential to monitor the emergence and spread of resistance to these partner drugs.

**Fig. 1:**
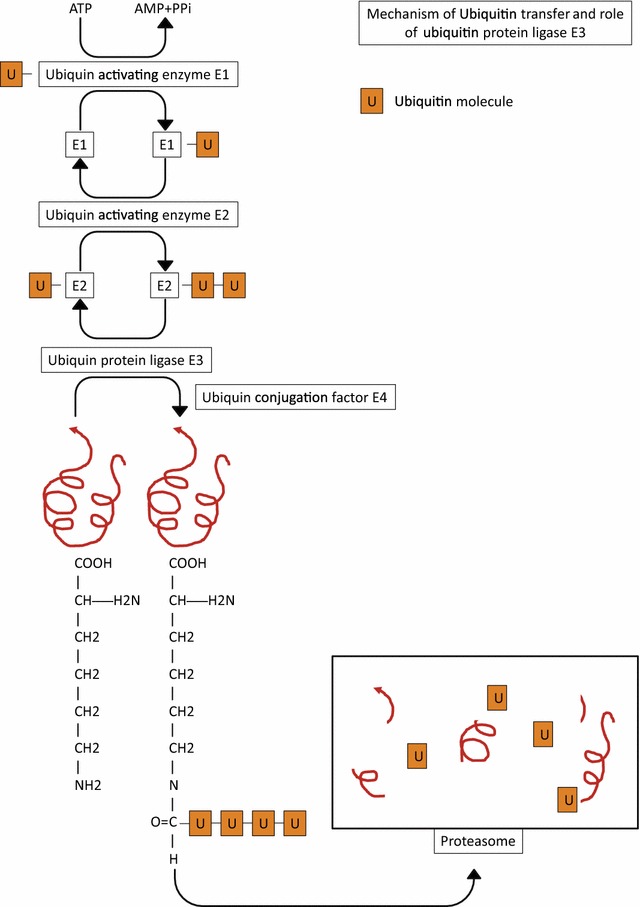
Mechanism of the ubiquitin pathway and the transfer role of the ubiquitin protein ligase E3

The ubiquitylation pathway is one of the major pathways used by all eukaryotic organisms to regulate protein abundance and protein activities in their cells. The molecular modification of proteins by ubiquitin, called ubiquitylation, is one of the main changes in post-translational events that are essential to most cellular processes. Ubiquitin is a 76-amino-acid polypeptide that is preserved in eukaryotic organisms, including *Plasmodium* spp. A single ubiquitin may bond covalently to a lysine residue on a protein through a complex enzymatic cascade involving activating, conjugating and ligase enzymes. Several ubiquitins can be bound to lysine residues on a single protein. Furthermore, ubiquitins can be bound to each other in polyubiquitin chains. At least four ubiquitins are required to designate the protein to a proteasome. To bind those ubiquitins, a multi-enzyme complex containing an E1 ubiquitin-activating enzyme, an E2 ubiquitin-conjugating enzyme and the E3 ubiquitin-protein ligase is involved (Fig. 1) [9, 10].

Ubiquitylation begins with the use of ATP by the E1 enzyme to adenylate its carboxyl terminus, allowing a molecule of ubiquitin to be transferred on the E1 enzyme active-site cysteine and resulting in a thioester bond between the E1 enzyme and ubiquitin. Afterward, a trans-thioesterification occurs with the E2 enzyme, which carries out the transfer of the E1 enzyme ubiquitin to a conserved cysteine on the E2 enzyme. In the end, the E3 enzyme removes the E2 bonded ubiquitin to create an isopeptide bond between the C-terminal glycine on ubiquitin and the epsilon-amino group of the lysine residue on the targeted protein. The target substrate is specific to the E3 ligase enzyme. The E3 ligase enzyme must bind at least four residues for proteasome addressing.

Three types of E3 ligase enzymes are described as follows: the homologous to E6-associated protein C-terminus (HECT) ligases, really interesting new gene (RING) fingers and finally, U-box E3 ligases. The latter type of ligase plays a primordial role in substrate specificity.

The ubiquitylation pathway could be a possible target for anti-malarial compounds and might be involved in the resistance of malaria [11, 12]. Previous publications using a genome-wide association study (GWAS) showed mutations in the E2 ubiquitin-conjugating enzyme gene (PF3D7_1243700) and the HECT E3 ubiquitin ligase gene (PF3D7_0826100) in Senegalese isolates that showed pyrimethamine resistance [13]. Furthermore, an HECT E3 ubiquitin ligase gene polymorphism might be involved in quinine- and quinidine-reduced susceptibility [14]. Additionally, *P. falciparum* strains carrying the mutant D113N allele for the E3 ubiquitin-protein ligase (PF3D7_0627300) gene were found to be less susceptible in vitro to chloroquine and amodiaquine [15]. Ribacke et al. used population genetics approaches to identify genes under positive drug selection. One of them was the RING ubiquitine ligase with the D113N mutation. They proved no difference in growth between parasites bearing the mutation and those which do not, but the mutant strains were less susceptible to CQ and amodiaquine. Parasite clones were then matched in competition experiments with the drugs and those carrying the mutation out-competed the wild types clones, suggesting the involvement of D113N mutation as a major involvement in ubiquitylation cascade and adaptive response to drug pressure. Another study showed no significant association between the D113N mutation and ex vivo reduced susceptibility to most conventional anti-malarial drugs, except a trend with reduced susceptibility to piperaquine in *P. falciparum* Senegalese isolates, but the major limitations of this study was the low number of isolates and the geographical provenance of them [16].

The objective of the current study was to analyse polymorphisms of the E3 ubiquitin-protein ligase gene (PF3D7_0627300) in *P. falciparum* African isolates, more particularly the D113N mutation and to evaluate the association of this polymorphism with ex vivo susceptibility to chloroquine (CQ), quinine (QN), monodesethylamodiaquine (DQ), mefloquine (MQ), lumefantrine (LMF), piperaquine (PPQ), pyronaridine (PND), dihydroartemisinin (DHA), artesunate (AS) and doxycycline (DOX) to confirm previous data.

## Methods

### Patients and sample collection

In total, 215 *P. falciparum* isolates were successfully sequenced for the E3 ubiquitin-protein ligase gene and assessed for ex vivo susceptibility to anti-malarial drugs. They were collected from patients hospitalized in France with imported malaria from a malaria-endemic country from January 2015 to December 2016 and, more particularly, from African French-speaking countries such as Côte d’Ivoire, Cameroon, Central African Republic, Republic of Congo, Guinea, Burkina Faso, Togo, Gabon and Senegal. The samples were sent from different civilian or military hospitals of the French National Reference Centre for Imported Malaria network (Aix en Provence, Bordeaux, Marseille, Montpellier, Nice, Toulon and Toulouse) to the French National Reference Centre for Malaria (IRBA, IHU Méditerranée Infection Marseille). The parasitaemia, ranging from 0.005 to 9.5%, was estimated on thin blood smears stained by eosin and methylene blue using a RAL^®^ kit (Réactifs RAL, Paris, France). The diagnosis of *P. falciparum* mono-infection was confirmed by real-time PCR (LightCycler 2.0, Roche Group, Switzerland) as previously described [17].

### Drugs

CQ, QN, DHA and DOX were obtained from Sigma (Saint Louis, MO, USA). DQ was from the WHO (Geneva, Switzerland), and MQ was purchased from Roche (Paris, France). LMF was from Novartis Pharma (Basel, Switzerland), and AS, PPQ and PND were from Shin Poong Pharm Co. (Seoul, Korea). QN, DQ, MQ, DHA, AS, PPQ and DOX were first dissolved in methanol and then diluted in water to final concentrations that ranged from 6 to 3149 nM for QN; 1.9 to 1988 nM for DQ; 1.5 to 392 nM for MQ; 0.1 to 107 nM for DHA and AS; 1.9 to 998 nM for PPQ and 0.5 to 497 µM for DOX. CQ and PND were resuspended and diluted in water to final concentrations ranging from 6 to 3231 nM and 0.4 to 199 nM, respectively. LMF was resuspended and diluted in ethanol to obtain final concentrations ranging from 0.6 to 310 nM.

### Ex vivo assay

The susceptibility of the 215 isolates to the ten anti-malarial drugs was assessed without culture adaptation. A total of 100 µL of parasitized erythrocytes (final parasitaemia at 0.5% and a final haematocrit at 1.5%) was aliquoted into 96-well plates that were pre-dosed with a concentration gradient of anti-malarial drugs (CQ, QN, MQ, DQ, LMF, DHA, AS, PPQ, PND and DOX). The plates were incubated for 72 h in controlled atmosphere at 85% N_2_, 10% O_2_, 5% CO_2_ and 37 °C. The drug susceptibility assay was done using the HRP2 ELISA-based assay Malaria Ag Celisa kit (ref KM2159, Cellabs PTY LDT, Brookvale, Australia) as previously described [18].

Each batch of plates was validated using the CQ-resistant W2 strain (isolated in Indochina; obtained from MR4, VA, USA) in four independent experiments using the same conditions described below. The mean 50% inhibitory concentration (IC_50_) values for the chloroquine-resistant W2 strain for the different batches used over 2 years were 484 ± 40 nM for CQ, 388 ± 29 nM for QN, 97 ± 18 nM for DQ, 1.0 ± 0.4 nM for LMF, 26.3 ± 3.1 nM for MQ, 54.1 ± 5.4 nM for PPQ, 20.4 ± 3.4 nM for PND, 2.5 ± 0.4 nM for DHA, 1.5 ± 0.3 nM for AS and 11.5 ± 1.9 µM for DOX. A comparison of the W2 susceptibility data of the ten anti-malarial drugs between the different batches of plates indicated that there was no significant difference in the responses to antimalarial drugs over the 2 years (0.583 < p < 0.993). The polymorphic genetic markers *msp1* and *msp2* and microsatellite markers specific to *P. falciparum* were genotyped at least once a month to verify W2 clonality [19, 20].

The cut-off values for the reduced in vitro susceptibility or resistance were as follows: 100 nM (CQ), 800 nM (QN), 80 nM (DQ), 30 nM (MQ), 150 nM (LMF), 135 nM (PPQ), 60 nM (PND), 10.5 nM (DHA and AS) and 35 µM (DOX) [21, 22].

### Gene sequence polymorphism analysis

The total genomic DNA of each isolate was isolated using the QIAamp DNA Blood Mini Kit according to the manufacturer’s recommendations (Qiagen, Germany). The E3 ubiquitin-protein ligase gene (PF3D7_0627300) is a 1349 bp gene whose the -5′ end region was amplified by PCR using the following primer pairs: 5′-AAT-GGT-CCA-GAA-GAA-GAT-TAT-3′ and 5′-ATT-TCG-AAT-TAT-CTT-CTA-CAT-C-3′. The -3′ region was not sequenced because the objective of the study focused on the previous findings concerning the D113N mutation only [15]. The reaction mixture contained 200 ng of genomic DNA, 0.32 µM of each primer, 1× final of reaction buffer (750 mM of Tris–HCl, 200 mM of (NH_4_)_2_SO_4_, 0.1% (v/v) Tween 20 and stabilizer, pH 8.8), 2.5 mM of MgCl_2_, 200 µM of dNTP mixture and 0.2 U of Hot Diamond Taq^®^ polymerase (Eurogentec) in a final volume of 25 µL. The thermal cycler (T3 Biometra) was programmed as follows: 10 min at 95 °C, then 40 cycles of 30 s at 95 °C, 45 s at 45 °C, 90 s at 72 °C and a final extension step of 10 min at 72 °C. The purified amplicons were sequenced using PCR primers and the sequencing primer 5′-AAT-ACT-TAT-GAT-ATG-ACA-AGT-GA-3′ on an ABI Prism 3100 analyser (Applied Biosystems) according to the manufacturers’ instructions. The sequences were analysed using BioEdit, version 7 (Ionis Pharmaceuticals, California, USA) to identify the D113N mutation.

### Statistical analysis

All the statistical studies, dot plots and the Africa map were created using the R software (R Core Team, Vienna, Austria).

## Results and discussion

**Fig. 2:**
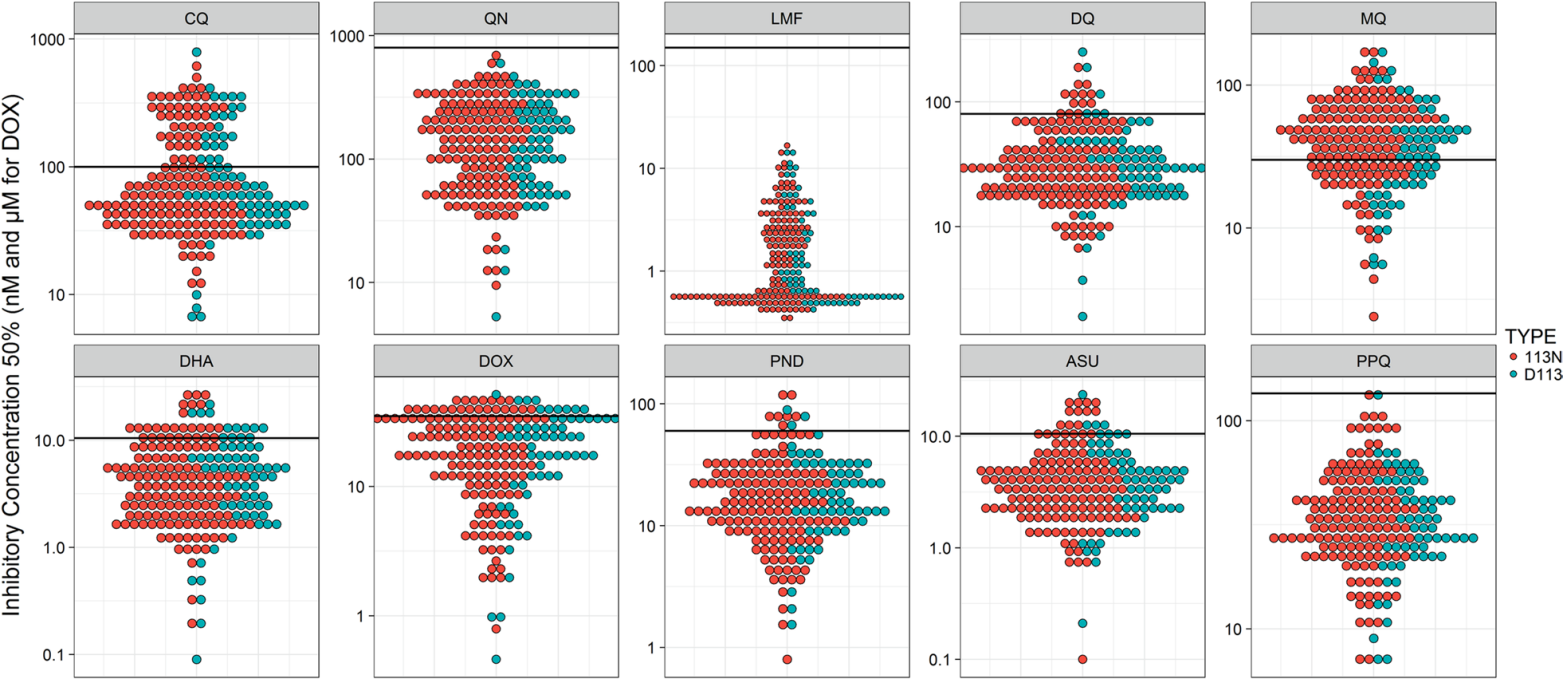
Dot plot of the IC_50_ total distribution of each *Plasmodium falciparum* isolate for chloroquine (CQ), quinine (QN), monodesethylamodiaquine (DQ), mefloquine (MQ), lumefantrine (LMF), piperaquine (PPQ), pyronaridine (PND), dihydroartemisinin (DHA), artesunate (AS) and doxycycline (DOX). Each dot is for an IC_50_ isolate, blue dots represent the wild D113 isolates and red dots the mutant 113N isolates. The black bar indicates the threshold for parasites with reduced susceptibility

The IC_50_ of the 215 isolates ranged from 6.27 to 500.6 nM for CQ, 5.29 to 690.11 nM for QN, 1.9 to 196.43 nM for DQ, 0.33 to 6.96 nM for LMF, 2.4 to 173.43 nM for MQ, 7.5 to 128.7 nM for PPQ, 0.8 to 122.96 nM for PND, 0.09 to 28.11 nM for DHA, 0.1 to 19 nM for AS and 0.46 to 44.89 µM for DOX. The global distribution of *P. falciparum* isolates IC_50_ is shown in Fig. 2.

Among the 215 isolates, 65.9% of the parasites showed reduced ex vivo susceptibility to MQ, 32.7% to CQ, 19,3% to DOX, 16,3% of DHA, 9.0% of DQ, 8,4% to AS and 4,7% to PND. The data concerning the decrease of the susceptibility to DHA and AS should be analysed with caution. The IC_50_ were estimated by a standard ex vivo test. Ring-stage survival assay (RSA), which is a better indicator of in vitro artemisinin resistance, should have been performed. Clinical resistance to artemisinin was manifested by an increase in the ring-stage survival rate after contact with artemisinin [23–25].

**Fig. 3:**
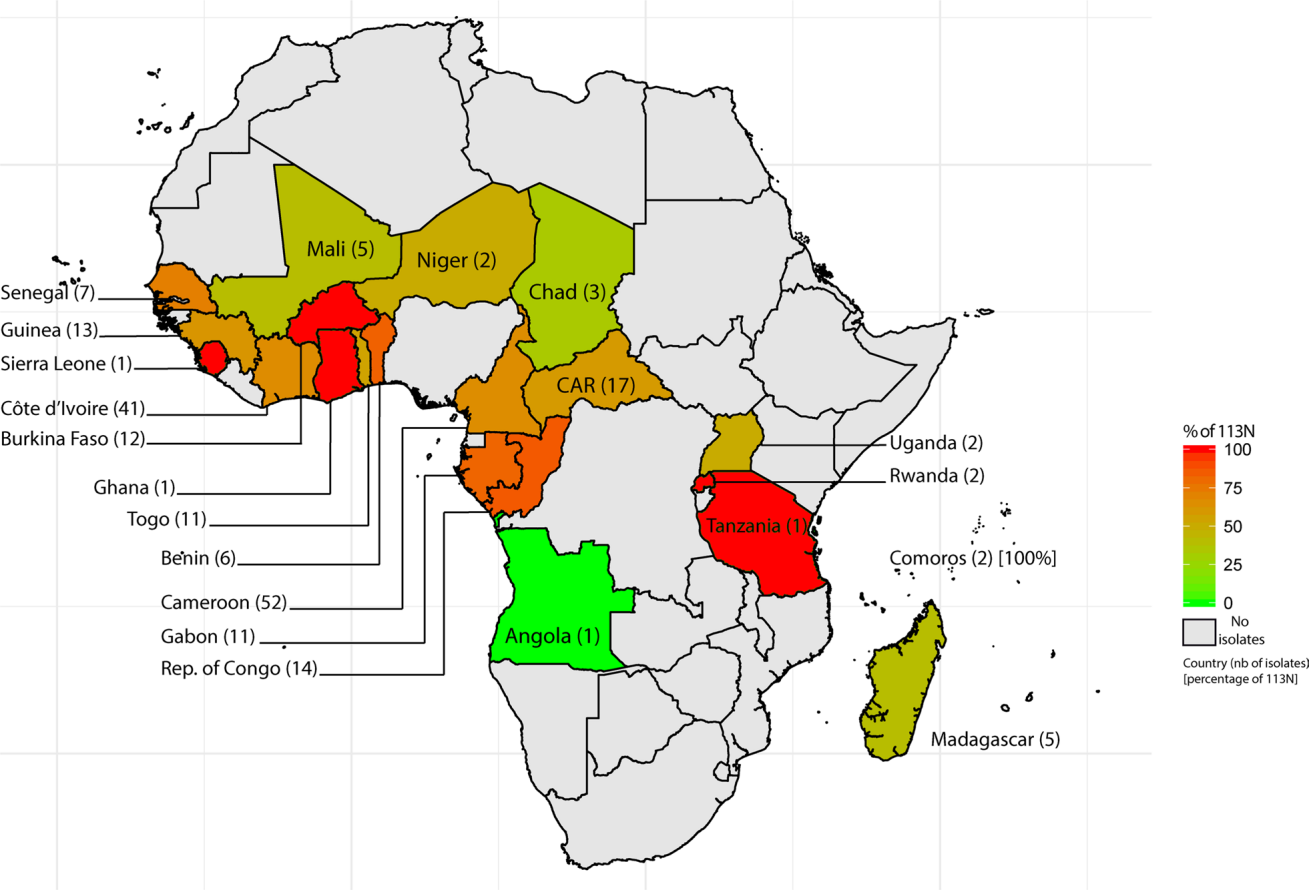
Repartition of the D113N mutation per its country of origin. The percentage of the D113N mutation in the E3 ubiquitin protein ligase gene (green to red coloration)

The D113N mutation of the RING E3 ubiquitin-protein ligase gene was present in 147 of the 215 samples (68.4%). The repartition of the D113N mutation per countries of origin is presented in Fig. 3. The D113N mutation was present in 69.6% of the isolates collected in 2015 (78 out of 112) and 66.9% in 2016 (69 out of 103). There was no significant difference between the 2 years and the proportion of mutant 113N isolates (p value = 0.7854, Chi squared test with Yates continuity correction).

A new mutation, E237D, was identified in a single isolate and conformed twice. This isolate showed the following IC_50_: 10.3 nM for PPQ, 28.7 nM for CQ, 121.4 nM for QN, 8.9 nM for DQ, 0.6 nM for LMF, 21.6 nM for MQ, 5.2 nM for PND, 5.3 nM for DHA, 5.8 nM for AS and 21.6 µM for DOX. This mutant parasite was susceptible to all the anti-malarial drugs tested.

The IC_50_ values for the ten anti-malarial drugs were not significantly different between the wild-type and mutant parasites (p values between 0.225 and 0.933) (Table 1). The distribution of the IC_50_ values for the ten anti-malarial drugs is presented in blue in Fig. 2 for the D113 wild-type parasites and in red for the 113N mutant parasites.

**Table 1:** Ex vivo susceptibilities of 215 African *Plasmodium falciparum* isolates to chloroquine (CQ), quinine (QN), monodesethylamodiaquine (DQ), mefloquine (MQ), lumefantrine (LMF), piperaquine (PPQ), pyronaridine (PND), dihydroartemisinin (DHA), artesunate (AS) and doxycycline (DOX) according to the D113N mutation in the RING E3 ubiquitin protein ligase (PF3D7_0627300) gene

Drug	Wild-type D113 (31.6%)		Mutated 113N (68.4%)		p value^c^
	Mean IC_50_^a^	Min and max^b^	Mean IC_50_	Min and max^b^	
CQ	75.5	[6.3–791.6]	75.1	[11.7–615.9]	0.933
QN	128.2	[5.3–631]	124.8	[9.5–690.1]	0.824
DQ	30.3	[1.9–251.5]	30.4	[6.3–196.4]	0.830
MQ	32.9	[5.5–173.4]	40.2	[2.4–172.6]	0.225
LMF	1.33	[0.4–15.1]	1.35	[0.33–16.7]	0.532
PPQ	31.9	[6.8–128.7]	34.0	[7.5–127.5]	0.398
PND	17.1	[1.67–89.2]	15.7	[0.8–123.0]	0.896
DHA	4.02	[0.09–21.1]	3.90	[0.2–28.1]	0.646
ASU	3.64	[0.21–23.6]	3.57	[0.1–21.2]	0.851
DOX	16.8	[0.46–51.1]	16.4	[0.79–49.9]	0.463

^a^Geometric mean inhibitory concentration 50%

^b^Minimum and maximum value for each drug

^c^p values were determined by the Student t test test

**Fig. 4:**
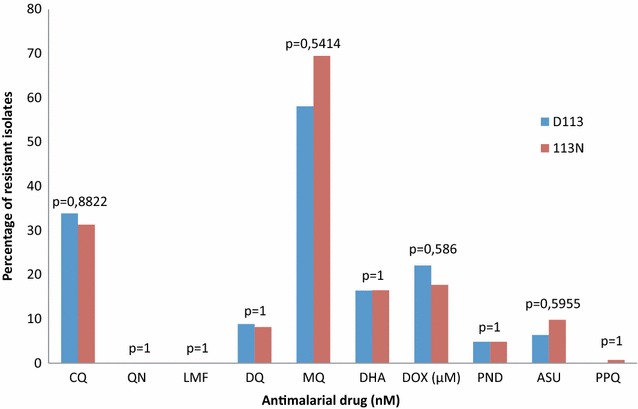
Comparison of the prevalences of *Plasmodium falciparum* isolates with reduced susceptibility to chloroquine (CQ), quinine (QN), monodesethylamodiaquine (DQ), mefloquine (MQ), lumefantrine (LMF), piperaquine (PPQ), pyronaridine (PND), dihydroartemisinin (DHA), artesunate (AS) and doxycycline (DOX) according to the D113N mutation in the E3 ubiquitin protein ligase gene. p values were determined using Fisher’s exact test

The isolates were categorized as susceptible or presenting ex vivo reduced susceptibility to the different anti-malarial drugs. There was no significant difference between the proportion of D113 wild-type parasites and 113N mutated parasites showing reduced ex vivo susceptibility to the different drugs used (p values between 0.541 and 1) (Fig. 4).

Polymorphisms of the RING E3 ubiquitin-protein ligase gene seem to be uniformly distributed, regardless of the African origin of the *P. falciparum* isolates. Among the 215 isolates, 52 came from Cameroon, and 41 were from Côte d’Ivoire. The parasites were mutant in 65 and 66% of the isolates, respectively. Countries with a low number of collected samples ranging from 1 to 10 showed a mean of 66.9% of mutant parasites. Countries with a number of collected isolates above 10 (except Cameroon and Côte d’Ivoire) showed a mean of 74% for the 113N mutant strains.

The emergence of resistance to ACT in Southeast Asia [3, 4], even to the most recently commercialized DHA-PPQ in Cambodia [5–8], requires the identification of new molecular markers of resistance to ACT partner drugs for monitoring the emergence and spread of resistance.

Previous studies showed that *P. falciparum* strains carrying the 113N mutant allele for the E3 ubiquitin-protein ligase gene were less susceptible ex vivo to CQ and DQ, and that all the Senegalese isolates with reduced susceptibility to PPQ were mutants [15, 16]. Although the D113N mutation was found in a large number of samples (n = 147, 68.4%), this mutation was not found to be involved in the susceptibility modulation to common anti-malarial drugs. The distribution of the IC_50_ values for all ten anti-malarial drugs tested did not show a significant difference between the D113 wild-type and 113N mutant groups. The decrease in the ex vivo susceptibility to CQ and DQ previously observed in genetically modified parasites was not found in these field African isolates [15]. This could be explained by the use of cloned parasites in the study of Ribacke et al., while African field isolates were assessed in the present study. The difference between clones and field parasites may induce differences between in vitro development of mutations and reduced susceptibility and wild isolates showing different patterns of gene sequence regulation and expression. There was no significant difference in the proportion of parasites with decreased susceptibility to PPQ between the D113 wild-type and the mutated 113N *P. falciparum* strains in contrary to previous findings [16].

## Conclusion

The ubiquitylation pathway, a key in post-translational regulation in eukaryotes, could be a possible target for anti-malarial compounds [11, 12, 26]. However, the present data confirmed the absence of an association between polymorphisms in the RING E3 ubiquitin-protein ligase gene and the ex vivo susceptibility to common anti-malarial drugs in African *P. falciparum* isolates.

## Abbreviations

ACT: artemisinin-based combination therapy
AS: artesunate
CQ: chloroquine
DHA: dihydroartemisinin
DNA: deoxyribonucleic acid
DQ: monodesethylamodiaquine
Dox: doxycycline
HECT: homologous to E6-associated protein C-terminus ligases
LMF: lumefantrine
MQ: mefloquine
PND: pyronaridine
PPQ: piperaquine
QN: quinine
RING: really interesting new gene
WHO: World Health Organization
